# Do Behaviour Assessments in a Shelter Predict the Behaviour of Dogs Post-Adoption?

**DOI:** 10.3390/ani10071225

**Published:** 2020-07-18

**Authors:** Liam Clay, Mandy B. A. Paterson, Pauleen Bennett, Gaille Perry, Clive C. J. Phillips

**Affiliations:** 1 Centre for Animal Welfare and Ethics, University of Queensland, Gatton, Queensland 4343, Australia; mpaterson@rspcaqld.org.au (M.B.A.P.); c.phillips@uq.edu.au (C.C.J.P.); 2 Royal Society for the Prevention of Cruelty to Animals Queensland, Brisbane, Queensland 4076, Australia; 3 School of Psychology and Public Health, La Trobe University, Bendigo, Victoria 3552, Australia; pauleen.bennett@latrobe.edu.au; 4 Delta Society, Summer Hill, Sydney, New South Wales 2130, Australia; perrygaille@gmail.com

**Keywords:** dog behaviour prediction, dog behaviour problems, dog behaviour assessment, canines, animal shelters, dog post-adoption behaviour

## Abstract

**Simple Summary:**

In shelters it is usual to conduct standardised behaviour assessments on all incoming dogs. The information gathered from the assessment is used to identify dogs that are suitable for adoption and assist in matching dogs with suitable adopters. We investigated the predictive value of the standardised behaviour assessment protocol currently used in an Australian shelter for dog behaviour post-adoption. A total of 123 dogs, aged 1–10 years and housed in an animal care shelter, were assessed before they were adopted. The new owners of the dogs took part in a post-adoption survey conducted 1 month after adoption, which explored the behaviour of their dog in its new home. Regression analyses identified that friendly/social, fear and anxiousness identified in the shelter assessment significantly predicted corresponding behaviours post-adoption. However, behaviour problems, such as aggression, food guarding and separation-related behaviours, were not reliably predicted by the standardised behaviour assessment. We recommend that dog behaviour assessments in shelters are used only in conjunction with other monitoring tools to assess behaviour over the whole shelter stay, thus facilitating increased safety/welfare standards for dogs, shelters and the wider community.

**Abstract:**

In shelters it is usual to conduct standardised behaviour assessments on admitted dogs. The information gathered from the assessment is used to identify dogs that are suitable for adoption and assist in matching the dog with suitable adopters. These assessments are also used to guide behaviour modification programs for dogs that display some unwanted behaviours. For some dogs, the results may indicate that they are unsuitable either for re-training or for adoption. In these circumstances the dogs may be euthanised. We investigated the predictive value of a standardised behaviour assessment protocol currently used in an Australian shelter for dog behaviour post-adoption. A total of 123 dogs, aged 1–10 years and housed in an animal care shelter, were assessed before they were adopted. The new owners of the dogs took part in a post-adoption survey conducted 1 month after adoption, which explored the behaviour of their dog after adoption. Ordinal regression analyses identified that friendly/social, fear and anxiousness identified in the shelter assessment significantly predicted corresponding behaviours post-adoption. However, behaviour problems, such as aggression, food guarding and separation-related behaviours, were not reliably predicted by the standardised behaviour assessment. The results suggest that further research is required to improve the predictability of behaviour assessment protocols for more specific behaviour problems, including different categories of aggression and separation-related problems. We recommend that dog behaviour assessments in shelters are used only in conjunction with other monitoring tools to assess behaviour over the whole shelter stay, thus facilitating increased safety/welfare standards for dogs, shelters and the wider community.

## 1. Introduction

In Australia, the Royal Society for the Prevention of Cruelty to Animals (RSPCA) is a National, not-for-profit organisation that accepts approximately 46,000 dogs per year [1]. A 2014 study [2] found that these dogs, most of which were adult, were most commonly admitted after being collected by local council officers as strays (34%). Others were presented by members of the public as strays (24%), owner surrenders (19%), or euthanasia requests (4%), with a small number being brought in by Humane Officers, employees of the RSPCA tasked with rescuing animals from situations where their welfare may be compromised (6%). Other studies have shown that relinquishment reasons are usually human-related (unwanted, changed circumstances, financial, owner’s health, household problems) but medical issues and behavioural problems also lead people to relinquish their dog [3,4,5,6,7,8,9,10]. In the Australian study [2] most dogs were either reclaimed (32%) or adopted (43%), with 14% euthanised. Reasons for euthanasia were dog behaviour (53%), dog health (23%), and owner requested (20%). If euthanased for behavioural reasons, it is likely that the dog displayed severe aggression, fearfulness and/or escaping behaviour.

Many shelters attempt to identify behavioural problems by continually monitoring behaviour and by formal behaviour assessments (BAs) while dogs are in care [11,12,13]. The behaviour assessments aim to identify behaviours that may cause problems in the dog’s future home, and to give an overview of the dog for potential adopters [14]. However, their ability to predict future behaviour or behavioural issues is questioned [15]. There is a concern that dogs that appear aggressive during a BA are being unnecessarily euthanased because they would not necessarily be aggressive in a home environment, and that non-aggressive dogs may be adopted out only to become aggressive at a later stage in the new home.

Life in a shelter is stressful and traumatic for dogs due to sensory overstimulation, social isolation, change/loss of control of daily routines and the novelty of the environment [14,16,17]. Stress has wide-ranging impacts, including on cognitive ability, behaviour and the dogs’ emotional state [18,19,20]. Therefore, a standardised BA conducted in shelters may not provide an accurate representation of the normal behaviour of the dog in a more stable and settled home environment.

Research conducted by Mornement et al. [14] in Australia compared the results of a Behaviour Assessment for Re-homing, K9′s (B.A.R.K.), administered in shelters, with results of a post-adoption survey. They reported that the only predictable outcomes were friendliness and fear-related behaviours. However, other behaviours, in particular aggression and food guarding, are rare post-adoption; Mohan-Gibbons [21] found that only six out of 96 adopted dogs were reported to display at least one incident of food guarding in the first 3 weeks, and at 3 months the adopters reported no food guarding behaviours at all. There was no evidence in this study, or a subsequent study [22], that food guarding increased return of the dogs to the shelter. In addition, injuries to staff, volunteers and adopters were rare and did not change if the food guarding test was omitted from the assessment.

‘Time alone’ tests have been used to identify dogs with separation-related behaviours [23]. Separation causes dogs to exhibit anxiety when away from owners or people in general; it is expressed as vocalisation, destruction of their environment, excretion, drooling, attempting to escape and depression-like responses [24,25]. Most shelters include a time alone test in their BA, during which the dog is placed alone in an unfamiliar room and observed for up to 10 min [23]. Dogs with separation-related anxiety spend the majority of the time vocalising, orienting to escape, panting and engaging in destructive behaviour.

Despite the current controversy about the use of BAs in shelters to gain an understanding of a dog’s behaviour and to identify any major or minor behavioural problems, we consider that assessments still have a role to play [26]. They can be used to identify stable behaviours. To further our understanding of how well BAs can predict dog behaviour in adoptees’ homes, we aimed to identify whether the standard BA protocol conducted at a Queensland shelter 5 days after admission predicted behaviour in adopters’ home environment, as assessed 1 month post-adoption.

## 2. Materials and Methods

### 2.1. Ethical Approval

This study was conducted with the approval of The University of Queensland Human Ethics Committee (2017000044). The RSPCA Animal Welfare and Ethics committee approved the use of data from the RSPCA Queensland survey of adoptees and behaviour assessment data.

### 2.2. Subjects

The dogs used in the study were housed at the RSPCA Queensland Animal Shelter at Wacol. Before inclusion in the experiment, dogs were assessed by a veterinarian and identified as having no apparent medical problems. Upon admission to the RSPCA, behaviour profiles were completed by the owners for owner-surrendered dogs (these were not available for stray dogs). Each dog was then evaluated by an RSPCA behaviour assessor using the RSPCA Qld. behaviour assessment 5 days after admission [13]. Data were collected from 955 dogs. Of the 955 owners that adopted these dogs, 125 were successfully contacted later and completed a post-adoption survey (14% response rate). Two owners initially agreed to participate in the study when contacted but later declined to take part.

### 2.3. Behaviour Assessment

A standardised behavioural assessment (Supplementary Materials) was conducted on all dogs during their stay at RSPCA Queensland by two staff (one Handler and one Observer/Rater) responsible for evaluating the dogs’ suitability for re-homing. These assessments were not able to be repeated due to staffing changes, therefore intra-rater and inter-rater reliability assessments were not possible. The assessments monitored the following behaviours: room exploration, behaviour when on a leash, sociability, tolerance, play behaviour with toys, tag (run and freeze), possessive behaviours, toddler and stranger interaction, time alone and social interactions with other dogs (RSPCA, 2012) [13]. The assessment comprised 11 different tests performed over a 15 min period, 10 have previously been described in detail [13]. The additional test ‘Response to a fake cat” is outlined in Supplementary Materials. The equipment used followed RSPCA Queensland’s protocol and included a 1.8 m leash, tennis ball, squeaky toy, rope, plastic hand on an extended pole, bowl, raw hide or bone, and combination of wet and dry dog food. At the conclusion of the behavioural assessment, animals were either deemed suitable for re-homing (*n* = 772), enrolled in a behaviour modification program (*n* = 133) or scheduled for euthanasia (*n* = 50). Decisions for behaviour modification and/or euthanasia were made by a professional review panel.

### 2.4. Behaviour Scoring by RSPCA Assessors

In each test, one RSPCA assessor rated the behaviour of the dog using binary occurrence of behavioural states (present or absent), except for the resource guarding test, which relied on a score by the assessor on an 8 point scale (Table 1). An overall score using the 11 tests was determined. All behaviours were assessed in each test using binary scoring (present or not) (Table 2)

### 2.5. Post-Adoption Phone Interview

Participants were asked when adopting a dog if they would agree to be included in a post-adoption phone survey. The survey was conducted by RSPCA customer service staff 1 month after adoption of the dog. The phone survey asked about the dog’s behaviour in the home environment and in different everyday situations (Supplementary Materials). It took approximately 10 min to complete and consisted of 36 multi-choice questions with the option to add additional information.

Participants rated the frequency of socialisation to owners and children, and behaviour with run and freeze play, an unfamiliar person, unfamiliar children, an existing dog, an unfamiliar dog, and interactions with cats, on a 5 point scale (1: moves towards you in a playful manner, 2: moves, leans, or looks away, 3: no response, 4: moves or leans away in a manner that concerns you, 5: moves towards you in a way that concerns you).

### 2.6. Statistical Analysis

Statistical analysis was conducted using Minitab 18. Behaviour data were first screened for errors and then transposed into percentage of occurrence in tests for descriptive analyses. Ordinal logistic regression analysis using a logit model was used to identify behaviours in the assessment that best predicted dog behaviour post-adoption.

## 3. Results

### 3.1. Descriptive Details

The sample included 123 companion dogs (males: 61, females: 62) over the age of 1 year and under 10 years. The sources for the 125 dogs were as follows: owner surrender (45%); transfer (17%); RSPCA officer intake (13%); stray (12%); return (6%); lost (5%); emergency ambulance intake (3%); and pound (1%). The majority of dogs in the study were mixed breeds (45%). Median time of stay in shelter was 55.5 days (range 3–114 days).

### 3.2. Behaviour Assessment (Table 3)

The number of dogs displaying the different behaviours during each test is presented in Table 3.

In Test 1, “Exploring the Room”, in the Exploration and Upon Call phases, dogs had a high occurrence of Friendly behaviour, with low occurrences of Anxious, Fear, and Arousal behaviours (Table 3). In Test 2, “Tolerance to Handling”, in all components the majority of dogs displayed friendly interactions with the assessor, with increases in Anxious behaviours in Stroke and Foot Sensitivity (Table 3). In Test 3, “Startle Response”, there was higher Avoidance, Fear, and Arousal in the Startle component, compared to the Recovery period, with a high occurrence of dogs displaying Friendly behaviours (Table 3). Recovery times varied between dogs, with 68% recovering within 5 s, 22% within 6–10 s and 3% taking over 10 s (7% of dogs did not exhibit as startle response).

In Test 4, “Toy Interactions”, there was a high occurrence of Play in all components of the test, with low instances of Fear and Anxious behaviour (Table 3). The component with the greatest number of dogs exhibiting Arousal was Rope interactions. In Test 5, “Response to Unusual/Predictable Stimulus”, there were high occurrences of Friendly behaviour in the Run and Freeze components but low levels of Anxious, Arousal and Fear behaviours (Table 3). In Test 6 (data not shown), “Resource Guarding”, dogs displayed a high occurrence of levels between 2 and 3 with wet (68.2%) and dry food (80%). There were low occurrences of levels 4–6 with bone (9.9%) or pig’s ear (7.43%).

In Tests 7 and 8 “Stranger Interactions” and “Toddler Interactions”, there were high occurrences of dogs displaying Friendly behaviour, with under 10% displaying Anxious or Displacement behaviours, Fear, or No Response towards the stranger (Table 3). Furthermore, there was only one dog that displayed Aggressive behaviour in each test. In Test 9, “Fake Cat”, there were high occurrences of Friendly behaviour towards the fake cat, with minimal dogs displaying other behaviours (Table 3). In Time Alone (Test 10), 51% of dogs displayed Separation-Related behaviours, 31.4% displayed no problematic behaviours and 18% displayed Anxious behaviours.

Finally, in Test 11, “Behaviour with Another Dog”, Friendly behaviours had the highest occurrence in dogs in all components of the test, with low levels of all other behaviours (Table 3). One interesting finding was the higher instance of Reactivity towards the opposing dog during the Walking component, which did not occur in the Circling or Nose to Nose components (Table 3).

### 3.3. Post-Adoption Behaviour

Only three participants no longer had the dog they had adopted. The remaining 120 participants still had their dog. With regard to the dogs’ living arrangements, 49% were indoor/outdoor dogs, 29% mainly indoors and 23% mainly outdoors.

Participants were asked how the dog responded to different situations (Table 4) with most owners outlining that the dog “moves towards the stimulus in a playful manner” and a low occurrence of the opposite response. In situations related to unfamiliar visitors and unfamiliar dogs, there were higher levels of “moves, leans or looks away”, “moves or leans away in a manner that concerns you”, and “moves towards in a way that concerns you” (Table 4).

In terms of interactions with cats, 93 (74%) participants did not answer, with 32 participants answering that their dogs interact with cats with 19% of dogs moving towards them in a playful/friendly manner, and under 3% displaying other behaviours. With respect to resource guarding, participants were asked whether they were concerned about their dog’s behaviour around food, treats, toys, and human food; over 90% reporting that there were no issues and under 10% saying there were issues (Table 5).

Participants were asked how their dog reacts to a loud noise or something else startling the dog. 37% ignored the question, 25% reported a mild startle response from their dog, 9% of dogs ran and hid, and 4% displayed a pronounced startle response. With dogs that were startled, participants were asked how long it took them to recover; 45% recovered immediately, 29% recovered within a few seconds, 15% recovered between 5 and 10 s, and 11% took longer than 10 s, avoided the situation and did not settle.

Participants were asked if they had ever left the dog alone, with 114 saying yes, and only nine saying no. Of the 114 participants that responded yes, 59% of dogs were left outside, 24% were left inside, 14% were allowed a combination of inside and outside, and 3% were left in a laundry or garage. Time spent alone ranged from 5 to 12 h (55%), 1–4 h (36%) and less than an hour (9%). Participants were asked whether their dog’s behaviour changed when they were preparing to leave, with 72% reporting no change and 28% some changes in behaviour. Participants were asked if any behaviours were of concern, with 80% saying no, and 21% saying yes.

### 3.4. Standardised Assessment Scores Verses Owner Surveys

Ordinal regression analyses were conducted to determine whether scores derived from the behaviour scores in assessment tests could predict behavioural traits in the new home using reported behaviour in the home environment as the dependent variable. Questions from the survey that called for a response along a 5-point scale were related to relevant tests in the assessment that measured interactions with the handler, children, strangers and dogs, as well as the startle response, response to usual stimulus, food items and time alone situations. The regression analyses found that friendly/social behaviours (scored in tests: Interaction with Assessor in exploration of room, Response to unusual/unpredictable stimulus, Stranger interactions, Behaviour with another dog) significantly predicted ‘playful/friendly manner’ behaviour post-adoption in interactions with owners, children, strangers, existing dogs and unfamiliar dogs (Table 6). Anxious behaviour (scored in the tests: Assessor in exploration of room, Response to unusual/unpredictable stimulus, Fake toddler doll and Behaviour with another dog) significantly predicted ‘Moving towards owner/children/stranger in a way that concerns you’ behaviour post-adoption with interactions with owners, unfamiliar child, running and freezing, and unfamiliar dog (Table 6). Fear (scored in the tests: Assessor in exploration of room, and Fake toddler doll) significantly predicted ‘Moves or leans away in a manner that concerns you’ post-adoption with interactions with owners, and children (Table 6). The remaining 13 post-adoption behaviours were not predicted by the standardised behaviour assessment protocol conducted at the shelter.

## 4. Discussion

The aim of this paper was to evaluate how well the standardised behaviour assessment (BA) protocol currently used in a Queensland RSPCA shelter predicted post-adoption behaviours. In general, the ability of the standardised BA protocol to predict specific behaviours post-adoption was only somewhat effective. It appears, then, that the standardised BA may, as previous authors have outlined [16], be useful as a tool for providing an overall measure of dog behaviour, particularly with respect to friendly, fearful, and anxious behaviour, but that it requires supplementation with other sources of information. However, our study was unable to adequately assess whether behavioural problems, specifically the identification of different categories of aggression, possessive behaviour (resource guarding), or separation anxiety, can be predicted from shelter assessments, since dogs displaying these behaviours were not rehomed.

There are several possible explanations for why the assessment was not more strongly predictive of our outcome measures. One constraint is that we cannot predict how an owner’s behaviour or personality, and other animals/individuals in the household, can influence/affect the dog’s behaviour post-adoption. Such effects may be substantial. Due to this, it may not be realistic to expect to be able to predict with accuracy behaviour over time.

A further explanation is that the standardised protocol may be inadequate as a tool to assess complex canine behaviours and behavioural problems either because of the structure of the assessment and/or its administration or due to the complex nature of such behavioural problems. We argue that the instrument is unlikely to be inadequately designed as it draws upon countless research studies and has been used and modified over many years [14,27,28,29,30]. The administration is also unlikely to have been inadequate, due to the standardised nature of the tests. Staff were trained and evaluated in the shelter, with the majority of the dogs in the large sample being assessed by the same individuals.

Another possible explanation is that due to the nature of canine behaviour, only some aspects of behaviour are stable [31,32]. Some aspects of canine behaviour may not be predictive in a single test, including aggression or other behaviour problems. Consistent with this idea was the number of new owners who reported their dog moving towards an individual in a way that concerned them, even though these dogs did not show these behaviours in the shelter assessment, or were not identified by shelter staff as displaying aggressive tendencies outside of the assessment. Dogs that displayed aggressive tendencies in the BA, or at other times during their stay at the shelter in the Queensland facility, were reviewed by a consultant for further testing. Such dogs were either then enrolled in a behaviour modification program or deemed to be unsuitable for adoption. Indeed, this study is similar to other studies in the area of canine behaviour assessment in shelters [12,21,22], where only dogs that did not show signs of aggression were made available for adoption and therefore included in the sample.

This suggests that there is a high possibility of a number of false negatives in the initial BA, which therefore is not offering a valid index of aggression. As seen in numerous studies, to reliably identify aggression and diagnose its causation is difficult, due to its infrequency and the nature of behavioural problems. Canine aggression is complex, and may be context specific [33]. The belief that one can assess a dog and diagnose it as aggressive is incorrect and should not be done. A specialist trained to identify and classify canine aggression would be in a better position to have a comprehensive understanding of physiology, behaviour and neurology, thus allowing a more nuanced diagnosis to be drawn [34]. Even in an assessment used primarily for identification of aggression, for example, the Dutch Socially Acceptable Behaviour (SAB) test, a portion of aggressive dogs remained undetected and the test was substandard for the assessment of types of aggression unrelated to fear [35]. This leads to the idea that fearful and anxious behaviours may be more stable and easier to detect than forms of aggression that can be motivated by numerous factors [17].

The final possibility is that canine behaviour may be predictable and the standard BA protocol used may be adequate at measuring certain categories of common/prominent canine behaviours (Friendly, Fearful, Arousal, Anxious), due to the common occurrence of these behaviour in everyday populations. However, due to the administration of the assessment after 5 days in the new environment, the tests may produce deceptive results. While many shelters maintain the highest standards of animal welfare, dogs still suffer from social isolation, abnormal sleep patterns, auditory pollution, olfactory overstimulation, and emotional stress, especially if individuals have no prior experience in shelters and do not habituate using positive coping mechanisms. The stressors that are inherent in any shelter may force some dogs to employ negative coping mechanisms (avoidance, inhibition or appeasement) as an outlet rather than displaying aggression [36,37]. This may especially be the case after surrender and over the first few days of entering the shelter, with some dogs likely to experience acute stress and social isolation [17]. Research into this area has found that shelter dogs showed more aggression when tested 2 weeks after being admitted to a shelter in comparison to 1–2 days after surrender [38]. Furthermore, only a few studies have studied the relationship of aggression with welfare standards for dogs [17,20] and whether the behaviour is due to environment stressors. Evidence in the literature suggests that stress can have an effect on cognitive function, negative emotional state and behaviour [18,19,20]. This implies that standardised canine BAs, timed incorrectly and used to make decisions about dogs (rehomed, trained or euthanised), may give false information to shelter staff.

Consistent with this possibility, recent studies into the test used to identify food resource guarding found the prevalence of issues post-adoption were low and that removal of the test did not increase the likelihood of food guarding in the new home [21,22]. The reason for this result can be identified in the complex aetiology behind food resource guarding. It is defined as the use of avoidance, threatening or aggressive behaviours by a dog to retain control of food or non-food items in the presence of a person or other animal [39]. It is not surprising that many dogs are so labelled in a shelter environment, due to the high occurrence of acute stress from sensory overload causing dogs to feel threatened and in turn aggressive. However, outside of the shelter environment, in a non-threatening and predictable environment, this reaction decreases. In addition, other types of aggression, such as territorial and maternal, remain very difficult to assess in shelters [33,40].

We advocate that shelters must look for a new approach that allows an improved ability to identify behaviour problems in a more stable environment. One such solution currently implemented in RSPCA Queensland shelters is the use of a foster care system, in which dogs that are unable to cope in the shelter are housed with foster carers until they are able to be adopted. This solution allows dogs to live in a stable environment with minimal exposure to stressors that may otherwise lead to the deterioration of the dog’s behaviour thus leading to behaviour problems. Furthermore, it allows shelters to house more dogs able to cope in the shelter environment, as well as individuals requiring behaviour modification and further testing of behaviour problems. In addition, RSPCA Queensland uses a qualified behaviourist to help to understand dogs that are identified in the behaviour assessment as having behavioural issues. The consultant conducts further tests to better identify the behavioural problems and implement behaviour modification programs with the use of qualified dog trainers. The dogs are constantly reviewed and evaluated to monitor progress over time.

However, implementing these solutions requires resources that most shelters do not have. Most shelters have financial, time and staff constraints that hinder them utilising such techniques. The authors understand that no one BA protocol has the ability to accurately predict every future behaviour, but these assessments can be used as one tool in conjunction with continual monitoring of behaviour and health of dogs in shelters, to gain an overview of the dog’s behaviour and identify dogs that require further testing or behaviour modification. Additionally, BAs can be used as monitoring tools to identify dogs not coping in the novel shelter environment. This, in conjunction with surrender information, veterinary monitoring and evaluations, in-kennel scoring from staff and volunteers, and behaviour modification should help develop a better system for shelters. To achieve this, continuous improvement and studies into dog behaviour in shelters are required.

## 5. Conclusions

Findings from this study suggest that a standardised behaviour assessment protocol used at an Australia shelter is a useful tool to predict some behaviours, mainly, friendly, fearful, arousal and anxious behaviours. However, in the predictability of behaviour problems, such as different categories of aggression or separation anxiety, it appears largely ineffective. This may be a result of the assessments being conducted in a highly stressful/novel environment where dogs experience many stressors in addition to lack of a human–animal bond, and then trying to use that information to predict home behaviour in a stable environment where supportive social bonds have formed. A thorough review of the protocol is recommended to identify any possible improvements, and care should be taken if the BA is the only tool used to identify a dog’s adoption suitability. However, using the BA as one tool in a toolbox of many others, including pre-surrender information, veterinary clinical assessments, monitoring in kennel and responses to training, may provide a more comprehensive picture of behaviour. Behaviour is multifactorial, requiring an in-depth understanding of multiple neurological and physiological processes. Therefore, continuous research and training in shelters together with ongoing support may help gain a better understanding of canine behaviour.

## Figures and Tables

**Table 1:** Resource guarding scoring system aimed at identifying possessive aggression by the dogs in defence of food.

Possession Level	Description
Level 1	Stops eating, wags tail loosely, and sniffs hand and looks to handler with soft eyes and relaxed body. Body language indicates no distancing behaviours.
Level 2	Continues eating, soft eyes, wags tail loosely, and body language indicates no distancing behaviours; typically a relaxed body stance/carriage.
Level 3	Continues eating but at a faster rate of intake. Body is slightly tense, particularly on human approaching the dog; tail wagging with an increased speed, especially on interaction with the dog and/or the food/treat. The dog blocks access to the food with their body (head and shoulder over the food and treat).
Level 4	The dog’s discomfort and behaviour starts to escalate. The dog glares, lifts its lip in a snarl, and/or produces a low growl. Increases eating speed, or with a treat the dog will whip its head away in an attempt to move it away from handler.
Level 5	Dog will carry the food item under a chair, bed, or into its crate, then growl on approach. If it cannot pick the food/treat up, it pushes the food bowl farther away. Dog freezes (stops eating or chewing), with whale eyes (exhibiting sclera) or direct stare, with or without lifting the lip in a snarl or other type of growl.
Level 6	Dog snaps but with no contact with fake hand. Level 5 behaviour usually continued but dogs move through the behaviours rapidly.
Level 7	Dog’s protectiveness increases with one or more rapid bites that touch the fake hand with quick and hard contact.
Level 8	Dog freezes with whale eyes or direct eye contact and biting aimed at the intruder even if they are at the perimeter of the room. At this level, it may be too dangerous to step into the perimeter to determine if the dog will bite or not.

**Table 2:** Behaviours evaluated in the Royal Society for the Prevention of Cruelty to Animals (RSPCA) Queensland canine behaviour assessment.

Behaviours	Definition
Play	Interacting with toys in social manner, may interact with handlers.
Friendly	May jump up on the person/dog licks person, dog nudges hand; play bow.
Social	Approaches and looks at assessor; stays with assessor making regular soft eye contact; low tail wagging, body relaxed, when assessor interacts may lower body.
Fearful	Cowers; runs away or avoids interaction, may tremble; tail tucked tightly, attempts to hide; at end of taut leash; mouth closed or panting excessively.
Anxious	Inability to settle and relax, distressed vocalisation, wide eyes, dilated pupils, excessive panting and licking, yawning and proximity seeking behaviour.
Arousal	Medium to hard mouthing of person; jump up and grab person’s clothing or body part; may mount person; inability to calm down; takes little to escalate the arousal levels.
Predatory behaviour	Sequence of behaviours that are associated with the catching and killing of another ‘animal’ for consumption, in this case a fake cat.
Reorienting	Changes direction away from stimulus.
Avoiding stimulus	Moves away from the stimulus.
Unresponsive	No behaviours change due to stimulus.
Aggression	Growls; shows teeth; snaps; directed stare; dilated pupils; attacks; bites
Displacement	The transfer of feelings or behaviour from their original object to a person or thing. Displacement behaviours include self-grooming, touching, stretching, yawning, displayed when an animal has a conflict between two motivations, such as the desire to approach an object while at the same time being fearful of that object.
Attracted to stimulus	Moving all the way to the end of the lead towards a stimulus until it is in full tension.
Appeasement	Individual attempts through appeasement displays to avoid injury by a dominant dog or human.
Reactive	Dogs respond with excessive reactions to a stimulus.
Separation related behaviours	Behaviours that are associated with being left alone; behaviours can include panting, pacing, excessive vocalisation, scratching at doors, excessive jumping, and damage.
Possessive behaviour	Aggression whilst guarding things (food bowls, rawhides, stolen, or found items, toys).

**Table 3:** Number of dogs (and %) exhibiting behaviour’s in the various test components in the behavioural assessment of shelter dogs (*n* = 123).

Test	Component	Behaviour															
		Friendly	Anxious	Fearful	Arousal	Appeasement	Aggression	Avoided	No Response	Displacement	Reorientated Away	Predation	Attraction to Stimulus	Reactive	Play	Possession	Separation Related Behaviours
1	**Exploring the room**	111 (85)	12 (9)	3 (2)	1 (1)	0	0	0	0	0	0	0	0	0	0	0	0
	Exploration																
	Upon Call	91 (70)	13 (10)	23 (18)	3 (2)	0	0	0	0	0	0	0	0	0	0	0	0
	**Tolerance to Handling**	73 (58)	19 (15)	10 (7)	2 (1)	21 (17)	0	0	0	0	0	0	0	0	0	0	0
	Collar																
2	Stroke	70 (56.5)	20 (15.6)	7 (5.6)	6 (5)	21 (16.7)	1 (0.6)	0	0	0	0	0	0	0	0	0	0
	Foot	68 (54.8)	15 (11.63)	6 (5.35)	12 (9.23)	23 (18.49)	1 (0.5)	0	0	0	0	0	0	0	0	0	0
3	**Startle response**	29 (24)	13 (10)	24 (19)	24 (19)	1 (1)	0	34 (27)	0	0	0	0	0	0	0	0	0
	Startle																
	Recovery	102 (82)	14 (11)	9 (7)	0	0	0	0	0	0	0	0	0	0	0	0	0
	**Toy interactions**																
	Tennis ball	0	0	5 (4)	11 (8.5)	0	0	0	16 (13)	0	0	0	15 (12)	0	75 (60)	3 (2)	0
4	Squeaky toy	0	0	3 (2.2)	10 (7.7)	0	0	0	14 (11.3)	0	0	0	28 (22.5)	0	68 (54.5)	3 (2)	0
5	Rope	0	9 (7.5)	9 (7)	19 (15.1)	0	0	0	4 (3)	0	0	0	8 (6)	0	75 (60)	0	0
	**Response to unusual/unpredictable stimulus**	87 (69.35)	16 (12.9)	12 (10)	0	0	0	0	3 (2.3)	1 (1)	0	0	0	0	0	0	0
	Run																
	Freeze	73 (58.25)	15 (11.7)	1 (1)	18 (14)	0	0	0	3 (2.6)	6 (4.5)	17 (14.3)	0	0	0	0	0	0
	**Stranger interaction**	105 (84)	9 (7.7)	3 (2)	0	0	0	0	5 (3.6)	3 (2.4)	0	0	0	0	0	0	0
7	Entry																
	Approach	98 (78.65)	7 (5.74)	8 (6)	1 (1)	0	0	0	1 (1)	9 (7.3)	10 (14.3)	0	0	0	0	0	0
	Leaving	68 (54)	0	0	0	0	1 (0.84)	0	0	15 (12.1)	41 (33.06)	0	0	0	0	0	0
8	**Fake toddler interaction**	93 (74)	8 (6.34)	9 (6.9)	4 (3.54)	0	1 (1)	0	0	11 (8.7)	0	0	0	0	0	0	0
	Approach																
	Leaving	71 (56.41)	0	0	0	0	1 (1)	0	0	9 (6.84)	43 (34.19)	0	0	0	0	0	0
9	**Fake Cat**	101 (81)	0	7 (5.36)	2 (1.7)	0	2 (1.7)	0	5 (4)	5 (4.34)	0	2 (1.7)	0	0	0	0	0
	Approach																
10	**Time alone**	0	22 (18)	0	0	0	0	0	39 (31.4)	0	0	0	0	0	0	0	64 (51)
	2 min																
11	**Behaviour with another Dog**	100 (79.84)	0	0	0	0	3 (2.48)	0	0	7 (5.52)	0	0	0	15 (12.16)	0	0	0
	Walking																
	Circling	88 (70.07)	6 (4.71)	2 (1.39)	8 (6.5)	0	9 (7.12)	0	2 (1.39)	4 (3)	0	0	7 (5.81)	0	0	0	0
	Nose-Nose	82 (65.93)	5 (3.9)	8 (6.45)	8 (6.23)	0	4 (3.15)	0	0	10 (14.33)	0	0	8 (6.23)	0	0	0	0

Test 6 resource guarding was not included in the table due to the different method of scoring of the behaviour.

**Table 4:** The percentage (%) of dogs (*n* = 120) displaying specific behaviours post-adoption.

Question	Moves towards in a Playful Manner (1)	Moves, Leans or Looks Away (2)	No Response (3)	Moves or Leans away in a Manner that Concerns you (4)	Moves towards in a Way that Concerns You (5)
Attention (Q5)	91.87	0.82	3.25	0.82	3.25
Children (Q7)	88.73	1.41	2.82	1.41	5.63
Run and freeze (Q8)	91.89	1.00	4.50	1.00	2.70
Unfamiliar visitors (Q9)	73.17	9.76	4.88	6.50	5.69
Unfamiliar children (Q10)	85.58	3.85	5.77	1.92	2.88
Existing dog (Q14)	84.62	5.13	0.00	2.56	7.69
Unfamiliar dog (Q16)	60.16	6.50	11.38	2.44	7.32

**Table 5:** The percentage (%) of dogs (*n* = 120) displaying possessive behaviour post-adoption.

Concern about Behaviour around Food, Treats, Toys and Human Food	No	Yes
Dog food	90.8	9.2
Treats	95.0	5.0
Toys	95.8	4.2
Human food	93.3	6.7

**Table 6:** Significant or trend level (*p* < 0.10) relationships between behaviours scored from the shelter behaviour assessment and responses in the post-adoption survey, analysed by ordinal logistic regression.

Behaviour	Test	Proportion Showing Behaviour in each Survey Category	Post Adoption	Coef	SE Coef	Z	*p*	Ratio	Lower	Upper
Friendly/social	1	0.91	Owners	2.50	1.45	1.73	0.05	12.21	0.71	208.88
	8	0.88	Children	2.68	1.20	2.23	0.02	14.65	1.39	154.41
	7	0.73	Stranger	1.06	0.55	1.94	0.05	2.89	0.99	8.46
	11	0.84	Existing dog	1.23	0.63	1.94	0.05	3.42	0.99	11.83
	11	0.60	Unfamiliar dog	1.42	0.63	2.27	0.02	4.14	1.21	14.16
Anxious	1	0.03	Owners	−1.43	0.79	−1.80	0.07	0.24	0.05	1.14
	11	0.07	Unfamiliar dog	−1.40	0.53	−2.62	0.01	0.25	0.09	0.70
	8	0.03	Unfamiliar child	2.38	1.02	2.34	0.02	10.83	1.47	79.46
	5	0.03	Run and freeze	−1.40	0.53	−2.62	0.00	0.25	0.09	0.70
Fearful	1	0.01	Owners	2.20	1.10	2.00	0.04	9.00	1.05	77.36
	8	0.01	Children	1.50	0.81	1.86	0.05	4.49	0.92	21.85

## Supplementary Materials

Supplementary Materials are available online at https://www.mdpi.com/2076-2615/10/7/1225/s1.
