# Supplementary material for: Do Behaviour Assessments in a Shelter Predict the Behaviour of Dogs Post-Adoption?
Source: Animals (Basel). 2020 Jul 18;10(7):1225. doi: 10.3390/ani10071225 (PMC7401658; doi:10.3390/ani10071225)
Supplement: Supplementary file 1 [file animals-10-01225-s001.zip › Appendix 1 edited 10.7.2020 submission.docx]

Appendix 1. RSPCA Standardised Behavioural Assessment (Clay, et al. 2019)

Test 1: Exploration of Room

Exploring the Room

The assessor entered the room, dropped the lead attached to the dog, and sat in the centre on a chair. Then, the observer started a timer and waited for 1 min without any interaction with the dog by either person.

Sociability to Assessor

At the end of exploring the room, the assessor called the dog to them in a friendly voice, remaining in the chair with no other body movement. If there was no response, a second attempt was made, and if still no response the assessor clapped their hands on their lap and said ‘come here’ in the direction of the dog, trying at least three times to call the dog to them. When the dog came (at the first, second, or third call), the assessor picked up the leash and then stroked the dog from the base of neck to tail three times. If the dog did not respond to the first, second, or third call the assessor approached the dog, picked up the leash, and gave the dog three strokes from the base of neck to tail. Following each stroke, the observer and assessor counted 10 s, with behaviours exhibited noted.

Test 2: Tolerance to Handling

There were three components to the test, touch sensitivity to collar, stroke, and feet. The assessor dropped the leash and held the dog’s collar. After 3 seconds, handler stroked the dog from head to tail. With the dog standing, the other assessor (in the standing position, or crouching if a small breed of dog) picked up the dog’s rear inside foot, then the front inside foot, then reached over its back to pick up its rear outside foot, and finally the front outside foot. Each foot was held for 2 s. After picking up all four paws in this manner, the assessor stood for 10 s with no dog interaction and finally removed the dog’s leash.

Test 3: Startle Response

There were two components: Startle response and recovery to stimulus. At the end of Test 2, the assessor created a loud sound using a book on a bench or desk (Startle response). Assessors recorded recovery.

Test 4: Toy Interactions

Three toys were used in this testing procedure: tennis ball, squeaky toy, and tugging rope. A tennis ball was shown to the dog and gently thrown across the room, and the assessor verbally engaged the dog in play. If the dog picked up the ball, the assessor waited to see if it returned to the assessor without encouragement. If it did not, the assessor encouraged the dog to bring the ball back by calling his/her name and saying “come”. If the dog still did not return, the assessor went to the dog.

In both situations, the assessor waited 10 s to see if the dog dropped the ball. If it did not, he/she asked the dog to “drop it”. If the dog did not respond, then a second command was given, “give”, and if necessary, a third attempt, “out”, was tried. If the dog did not respond to these commands, the assessor approached the dog carefully and removed the ball from the dog’s mouth. These steps were repeated for a second throw and, after completion, the assessor waited 10 s with no interaction before moving on to the next toy, the squeaky toy, and after that the tugging rope. The same sequence was used for each toy. After completing all three toys, the assessor moved on to the next test.

Test 5: Response to Unusual/Unpredictable Stimulus

The assessor gently moved the dog to the opposite end of the room and left it standing against the wall. Then, he gently moved one hand over its head, down toward the back to gently tap the rump area, and then ran across the room, laughing and waving arms, followed by suddenly stopping, folding his arms, and ignoring the dog. The tap, run, and freeze series was repeated a second time. The assessor waited for 10 s after the run and freeze, ignoring the dog, before moving onto the next test. The dog was then placed back on the leash.

Test 6: Resource Guarding

There were four components to the test: Wet food, dry kibble/biscuits, pig’s ear and bone. The assessor tethered the dog to the wall for safety reasons, and proceeded to show the dog wet canned food, smeared in a bowl. The bowl was then placed near the dog at the end of the leash perimeter, allowing the dog to begin eating for 2 s. The assessor then proceeded with a plastic hand, walking to the side of the dog while it was eating. Using the fake hand, the assessor patted the dog on the head, continuing to stroke down its back and body twice. The fake hand was then placed 5 cm in front of the bowl and moved around in a semi-circle. The hand was then placed on the inside edge of the bowl and moved around the edge of the bowl next to the dog’s face, without touching it. Finally, the bowl was pulled away from the dog using the fake hand. The bowl was then returned to the dog, which was observed for 10 s.

The assessor then gave the dog a pig’s ear or bone, depending on the dog’s food interest, and it was allowed to chew it for 30 s. The steps above with wet food were repeated; then, the assessor attempted to retrieve the food, asking the dog to “drop it”, “leave it”, or “give” before attempting to retrieve it by offering a new food that is novel.

Test 7: Stranger Interaction

There were three components to the test: entry, approach and exit of stranger. The assessor placed the dog on a leash as the observer exited the room and returned dressed in a reflective vest, large brimmed hat and using a walking stick. The observer entered the room, and bent down to extend an open flat hand as if to pat the dog on the head. The observer then talked to the dog normally and stopped for 3 s, allowing the dog to approach. If the dog approached, the observer patted the dog on the top of its head for 3 s. If the dog did not approach, it was observed for 10 s, with an emphasis on any interaction between the assessor and/or the observer.

Test 8: Fake Toddler Interaction

There were two components of the test: approach of the toddler doll and exit/removal of toddler doll. The assessor stood and held the dog’s leash while the observer exited the area and returned carrying a toddler doll simulating a small child. Once the toddler was within the leash perimeter from the dog, the observer placed the doll on the floor facing the dog, with the doll’s arm extended toward the dog. The assessor allowed the dog to approach if it desired. If the dog did not approach the observer, it was observed for 20 s. After this, the assessor picked up the toddler doll and walked back out of the room. The assessor allowed the dog to follow to the door or move away from stimulus.

Test 9: Fake Cat

The assessor stood and held the dog’s leash while the observer exited the area and returned carrying a fake cat as if it were a “real” cat. Once the fake cake was within the leash perimeter from the dog, the observer placed the fake cate on the floor facing the dog. The assessor allowed the dog to approach if he/she wanted to. However, if the dog did not approach the observer, the dog was observed for 20 s with the fake cat present.

Test 10: Time Alone

The assessor and observer removed the leash from the dog and left the room for 2 min, with a video camera in the front of the room monitoring behaviour and vocalisations. Then, the assessor and observer re-entered through the same door.

Test 11: Behaviour with Another Dog

There were three components to the test: walking parellel, circling activity, and nose to nose interaction. This test was conducted in a yard (10−20 m), allowing adequate space between the test dog and another dog. Each dog had an assessor, who interacted with their dog by giving treats and ignoring the other assessor and dog. The assessor had a short, 1 m, leash, so that the dog walked close to the assessor. At the start, both assessors walked parallel to each other, 5 m apart, with the dogs on the outside. If one or both dogs were reactive and pulled toward each other, the distance between the assessors was increased. If both dogs were relaxed and focused on their assessor, the assessors moved the dogs to an exercise circle. If the dogs did not breach a minimum distance of 5 m between them, they were introduced on opposite sides of a fence. There followed a circling activity, which required one assessor to stand still with their dog on no more than 1.5 m of leash while the other assessor and their dog completed a circle around the assessor. Assessors then swapped places and repeated the circling activity. If no adverse behaviours were displayed, the assessor in the middle of the circle remained at that location, ensuring that the only tension on the leash was from the dog. The other assessor identified the leash threshold of the dog in the centre and moved close enough to allow the dogs to be nose to nose, also ensuring that the only tension on their leads was caused by the dog pulling, not them pulling against the dog. Once the leads became loose, and the dogs stopped pulling against the assessor, the assessors took a step closer to each other, allowing the dogs to interact if they chose. Leashes remained loose. If there were signs of adverse reactions or aggression, dogs were separated by increasing the threshold.
