# Supplementary material for: Do Behaviour Assessments in a Shelter Predict the Behaviour of Dogs Post-Adoption?
Source: Animals (Basel). 2020 Jul 18;10(7):1225. doi: 10.3390/ani10071225 (PMC7401658; doi:10.3390/ani10071225)
Supplement: Supplementary file 1 [file animals-10-01225-s001.zip › Appendix 2 13.7.2020 submission.docx]

Appendix 2

Telephone Survey Script for Adopters

# Introduction:

Hello, my name is ………. from the RSPCA – you recently adopted …….. from our Wacol Animal Care Campus and you might remember we asked if you would mind completing a survey on how …….is settling into your home. The information you are able to provide us will be really valuable to help us improve our current procedures. We anticipate the survey to take xxx minutes - is now a convenient time?

If No – Thank you.. would there be a more convenient day / time that might work better for you?

If Yes – Thank you! Most questions are yes or no, and then some clarifying multiple choice options that I will provide you. I would be happy to repeat them for you as needed. Are you ready?

*If they say yes continue.*

1. Do you still have ………? Y N
   1. If no, may I ask what happened?
      1. Returned to RSPCA
      2. Surrendered to other shelter
      3. Gave away to a known person
      4. Gave away to an unfamiliar person
      5. Escaped
      6. Lost
      7. Deceased
      8. Other
2. If returned or gave away – can I ask the reason? Type reason in comment box
3. How long did you have xxxx in the home?

If longer than two weeks continue with survey if less than two weeks thank them for their time and offer any assistance in the future.

## If they still have the dog, or had dog longer than two weeks continue:

I am going to continue with the interview questions and provide you with some options – could you tell me which best describes their behaviour please?

1. Is your new dog
   1. Mainly indoors
   2. Mainly outdoor
   3. Indoor / Outdoor

5. How does the dog respond to your attention?

- 1. No response
  2. Moves, leans or looks away
  3. Moves towards you in a playful manner
  4. Moves or leans away in a manner that concerns you
  5. Moves towards you in a way that concerns you

If d, what do they do? (text box)

6. Do you have children? (Or do children visit the home) Y N

- 1. Under 5 years
  2. Between 5-10 years
  3. Between 10-16 years

7. How does the dog respond to them?

- 1. No response
  2. Moves, leans or looks away
  3. Moves towards them in a playful manner
  4. Moves or leans away in a manner that concerns you
  5. Moves towards them in a way that concerns you

If d, what do they do? (text box)

8. Has anyone tried to play a running game around/with your new dog? Y N

If so, how do they play?

- 1. No response
  2. Moves, leans or looks away
  3. Moves towards them in a playful manner
  4. Moves or leans away in a manner that concerns you
  5. Moves towards them in a way that concerns you

If d, what do they do? (text box)

9. How do they respond to unfamiliar adult visitors at home?

- 1. No response
  2. Moves, leans or looks away
  3. Moves towards them in a playful manner
  4. Moves or leans away in a manner that concerns you
  5. Moves towards them in a way that concerns you

If d, what do they do? (text box)

10. How do they respond to unfamiliar children at home?

- 1. No response
  2. Moves, leans or looks away
  3. Moves towards them in a playful manner
  4. Moves or leans away in a manner that concerns you
  5. Moves towards them in a way that concerns you

If d, what do they do? (text box)

11. We gave you a handout on introducing your new dog to the children did you find it useful? Y N did not read

12. Do you have another dog? Y N

13. Can you tell us about how your new dog behaved when they first met?

- 1. No response
  2. Moved, leaned or looked away
  3. Moved towards them in a playful manner
  4. Moves or leans away in a manner that concerns you
  5. Moved towards them in a way that concerned you

If d, what did they do? (text box)

14. Can you tell us about how your existing dog behaved when they first met?

- 1. No response
  2. Moved, leaned or looked away
  3. Moved towards them in a playful manner
  4. Moves or leans away in a manner that concerns you
  5. Moved towards them in a way that concerned you

If d, what did they do? (text box)

15. We gave you a handout on introducing your new dog to your existing dog was it useful? Y N did not read

16. How does your dog behave if they see an unknown dog outside your home?

- 1. No response
  2. Moves, leans or looks away
  3. Moves towards them in a playful manner
  4. Moves or leans away in a manner that concerns you
  5. Moves towards them in a way that concerns you

If d, what do they do? (text box)

1. Are you concerned about your dog’s behaviour around their food

If yes can you tell me what happens (text box)

1. Are you concerned about your dog’s behaviour around food toys

If yes can you tell me what happens (text box)

1. Are you concerned about your dog’s behaviour around human food

If yes can you tell me what happens (text box)

1. Do you have a cat? Y N
2. If yes how would you describe your new dog’s initial behaviour towards the cat?
   1. No response
   2. Moved, leaned or looked away
   3. Moved towards them in a playful manner
   4. Moves or leans away in a manner that concerns you
   5. Moved towards them in a way that concerned you

If d, what did they do? (text box)

1. Did you find the informational handout on introducing your new dog to your cat useful?
   1. Yes
   2. No
   3. Did not read
2. If the new dog hears a loud noise or something startles them, do they
   1. Ignore it (No response)
   2. Mild startle
   3. Pronounced startle
   4. Run and hide
   5. Other (text box)
3. Afterwards do they settle down again?
   1. Immediately
   2. Within a few seconds
   3. 5-10 seconds
   4. Longer
   5. They don’t settle
   6. They continue to avoid the location of the sound / startle
   7. Other
4. Have you ever left your new dog alone? Y N
5. If yes, where did you leave him alone?
   1. Inside the house
   2. in a crate
   3. In the laundry or garage
   4. Outside in the yard
   5. Other
6. How long did you leave the new dog alone for?
   1. Less than an hour
   2. 1-4 hours
   3. 5-8 hours
   4. 9-12 hours
   5. Other
7. How did they behave when you were preparing to leave?
   1. No change in behaviour
   2. Change in behaviour – please describe (text box)

Was their behaviour while you were away a concern?

If yes, why (text box)

1. Did you find the information on teaching your dog to stay home alone that we gave you useful? Y N Did not read

Thank you for participating in the study. If we at the RSPCA can assist you in the future please let us know.
